# Cryotherapy for oral potentially malignant disorders

**DOI:** 10.6026/973206300191377

**Published:** 2023-12-31

**Authors:** Ashutosh Pandey, Sukirat Kaur, Albert Ashem, Shivangni Sarin, Rakesh Kumar Sahu, Braina P. Jadav, Dhaval Niranjan Mehta, Ramanpal Singh Makkad

**Affiliations:** 1 Department of Dentistry, Anugrah Narayan Magadh Medical College and Hospital, Gaya, Bihar, India; 2 Department of Public Health Dentistry, Genesis Institute of Dental Sciences and Research, Ferozepur, India; 3 Department of Oral Medicine and Radiology, Dental College, RIMS, Imphal, Manipur, India; 4 Department of Oral & Maxillofacial Surgery, Army College of Dental Sciences, Secunderabad, India; 5 Department of Oral Medicine and Radiology, New Horizon Dental College and Research Institute Sakri, Bilaspur, Chhattisgarh, India; 6 Department of Oral Medicine and Radiology, Ahmedabad, India; 7 Department of Oral Medicine and Radiology, Narsinbhai Patel Dental college and Hospital, Sankalchand Patel University, Visnagar, Gujarat, India; 8 Department of Oral medicine and Radiology, New Horizon Dental College and Research, Institute, Sakri, Bilaspur, Chhattisgarh, India

**Keywords:** Cryosurgery, LASER, OPMDS

## Abstract

Four surgical treatment modalities namely cryosurgery, scalpel and blade surgery, diode LASER surgery and CO2 LASER surgery in the management of oral potentially malignant disorders (OPMDs) in terms of healing outcomes post operatively and recurrence is evaluated. The study included sixty outpatients whose biopsies revealed OPMDs (oral lichen planus, homogeneous leukoplakia, non-homogenous leukoplakia and erythroplakia). There is decrease in post-operative pain and oedema in all four treatment categories at one week follow up and two week follow up. It was observed that pain was low in cryosurgery treatment category at day of surgery as well as at one week of follow up as compared to diode LASER and CO2 LASER. Observations from the study highlights that all four surgical modalities used in this study were effective for treatment of OPMDs, and the overall summation of the results of the study showed that cryotherapy seems to offer better clinically significant results than laser therapy.

## Background:

Cancer of oral cavity is one of the most serious issues of community-based health around the globe public health concern around the world [1,2,3]. One-third of the cases (37.5 percent) were found only in Asia. Scientists coined the phrase "premalignant lesion" to describe a disease that, if left untreated, could grow causing "cancer" [4,5]. Oral premalignant lesions (OPMLs), which affect about 3% of total human population, are a specific target for prevention of cancer of oral cavity [6,7]. The importance of pre-cancer lesion like oral leukoplakia, oral erythroplakia, or oral erythro leukoplakia arises from the large number of patients where a biopsy reveals dysplasia or similar "frank cancer [8-9]. There is a progressive histopathological sequence which can be graded as regular, hyperplastic as well as carcinoma in situ during the progression of premalignant lesion to the malignant lesion [10,11]. Oral pre-malignant lesions can be identified and managed with a visual inspection and are conveniently accessible for further testing including microscopy and biopsies [12,13].Early diagnosis of abnormalities reduces mortality and morbidity, but prolonged identification, particularly in places with the highest incidence rate, lowers the chance of survival, despite modern treatment procedures [14,15]. Pre-cancerous lesions of the oral cavity include leukoplakia of oral cavity, erythroplakia of oral cavity, oral sub-mucous fibrosis, condition of actinic keratosis, condition of discoid lupus erythematosus, condition of lichen planus of oral cavity, and condition of reverse smokers, all of which are referred to as "potentially malignant illnesses (OPMDs) [16, 17]. It has been documented that around 13% of these abnormalities of oral cavity get transformed into cancer of oral cavity [18,19]. Elimination of contributing variables such as tobacco use, smoking, discontinuing betel quid, and reduced or complete abstinence from alcohol consumption is the initial step in managing OPMDs [20,21]. Total quitting smoking can have a significant impact on the prognosis. The severity of the lesion, especially its location, dimensions, and position, together with any accompanying dysplasia, will also determine the course of treatment [22, 23]. Numerous medicinal and surgical therapy techniques are used to control OPMDs. Reversing or completely eliminating the alterations to the oral mucosa is the aim of OPMD treatment. The medical line of care for OPMD comprises topical as well as systemic medicines such as vitamin A and retinoid, lycopene, ketorolac, systemic beta-carotene, topical and systemic corticosteroids and local bleomycin, with varying degrees of efficacy [24,25]. While retinoid, or vitamin A, has been utilized as a medical treatment, there is not much scientific data to support its efficacy in lowering the rate of cancer transformation and relapse [13-15]. Traditional surgery using scalpel and blade and LASER (Light Amplified Stimulate Emission of Radiation) surgery are among the surgical treatment techniques that have been recommended for the management of OPMDs [16- 17]. The location and size of the disease restrict conventional operations. A straightforward, secure, tried-and-true method of treating OPMDs and some benign and precancerous lesions of the oral cavity is cryosurgery. Compared to traditional surgery, lasers offer a multitude of advantages, including targeted excision, easy achievement of homeostasis, and less discomfort and oedema after operation [18,19]. Although cryotherapy is being successfully utilized in management of different medical surgery, but its use in management of OPMDS is still being a field of research. No study has compared cryotherapy with other surgical modalities for OPMDS namely conventional scalpel blade surgery and LASERs [20-23]. Hence, four surgical treatment modalities namely cryosurgery, scalpel and blade surgery, diode LASER surgery and CO2 LASER surgery in management of oral potentially malignant disorders (OPMDs) in terms of healing outcomes post operatively and recurrence were evaluated.

## Methods and Materials:

The study included sixty outpatients whose biopsies revealed OPMDs (oral lichen planus, homogeneous leukoplakia, non-homogenous leukoplakia, and erythroplakia). The benefits and drawbacks of surgery were discussed with research participants. Blood examinations were performed on the patients, comprising clotting times, complete hemograms, bleeding times and random blood sugar tests. Patients with current cancer and those with OPMDs that reduced in size after habit discontinuation were not included in the study. There were 20 women and 40 males in the study sample, having a mean age of 35.5 years. Sixty patients were divided into four categories at random, with fifteen patients in each category. All the groups received any of the four treatment modalities (Table 1). A questionnaire was used to assess each patient, gathering information about their prior medical history, surgical history, and dental histories. An intraoral as well as extraoral assessment was also conducted. 2% lignocaine was mixed with 180000 epinephrine was injected into the surgical site for each group as a local anesthetic. In category 1: A scalpel and 15 no blade was used to mark an incision at the circumference of lesion in the form of triangular incision with base at posterior border and apex at anterior portion of lesion. The lesion along with tissue was retracted with a tissue forceps and complete excision was carried out. It was followed by suturing. In category 2: Local anesthesia was applied while a 4 watt diode laser system operating at 970 nm was employed continuously. Utilising a surgical marker, the lesions were located and their boundaries noted. The lesion was totally ablated when the treatment was administered in contact mode. In category 3: A application directly of nitrous oxide cryoprobe with a temperature of -65 to -85 degrees Celsius was used to carry out the cryosurgery process. After the ice ball formed, the cryoprobe was administered for 45-60 seconds while the tissue was given time to fully thaw before the probe was reapplied. For a minimum of 2-3 freeze-thaw cycles, the cryoprobe was used. After applying the probe to neighbouring locations one after the other, a quick frozen area-filled spatial overlap was created. In category 4: Surgery was conducted using a 10.6 μm wavelength CO2 laser beam. The targeting beam was employed, with a typical spot size of 1 mm, and the power output was kept within the customary standard range of 5 to 15 watts on pulsed/continuous mode. The ablation was carried out in a non-contact mode by manoeuvring a slightly defocused point of CO2 laser over the lesion until full evaporation while it reached sub-mucosa. The area of tissue to be ablated was first marked with a margin of around 3mm in a pulsed mode. To cure extensive lesions, the mapping approach had to be applied multiple times. On the day of the procedure, the bleeding and pain levels of each patient in the four categories were evaluated. A visual analogue scale was used to assess pain, with 0 and 10 representing the extremes and representing "no pain" and "maximum pain imaginable," respectively. By noting whether spontaneous bleeding occurred or not, bleeding was evaluated. Evaluation of oedema consisted of comparing the region of the wound to the anatomical region of the contralateral side to see whether asymmetry existed. Infection, scar development, and slough were assessed solely by visual inspection. During the 1st and 2nd postoperative week, patients had follow-up appointments to monitor pain, oedema, slough development, and infection. Evaluations of the development of scars and lesion reappearance were conducted three months and six months after surgery. Infections or problems during and after surgery were later identified.

## Statistical analysis:

The frequency distribution for each research parameter (categorical variable) that was to be compared between the four categories was given in terms of number and percentage. The distribution and association of the research variables among the four categories at each time interval were compared using the Chi-Square test. The pain scores' mean and standard deviation (SD) were calculated, and the one-way ANOVA test was used to compare the groups' scores. Tukey's HSD test was then used as a post hoc analysis. P<0.05 was used as the significance level (P-Value) threshold.

## Results:

In this study, 20 cases of homogenous leukoplakia, 14 cases of non-homogenous leukoplakia, 18 cases of oral lichen planus and 8 cases of erythroplakia were included (Table 2). There is decrease in post-operative pain in all four treatment categories at one week follow up and two week follow up. It was observed that pain was low in cryosurgery treatment category at day of surgery as well as at one week of follow up as compared to diode LASER and CO2 LASER. The findings were statistically significant (p=0.001). It was observed that post-operative pain at two weeks of follow up was comparable in all treatment modalities. VAS values for pain at day of surgery was 2.9± 0.13, 2.1± 0.01, 1.6± 0.34 and 2.0± 0.12 in scalpel-blade surgery, Diode LASER , cryotherapy and CO2 LASER respectively. Similarly VAS values for pain at one week follow up was1.6±0.02, 1.4±0.04, 1.1± 0.31 and 1.5± 0.14 in scalpel - blade surgery, Diode LASER, cryotherapy and CO2 LASER VAS scores. VAS values for pain at two week follow up was 0.7±0.04, 0.5±0.01, 0.2± 0.02 and 0.4± 0.03 in scalpel- blade surgery, Diode LASER, cryotherapy and CO2 LASER (Table 3). There was maximum pain in scalpel blade surgery at day of surgery and one week follow up.

There was low proportion of patients with oedema in cryosurgery group as compared to Diode LASER, scalpel blade surgery and CO2 LASER, at one week follow up. The findings were significant statistically (0.001). There was decrease in oedema in all treatment modalities at two week follow up. The proportion of oedema in at two week follow up in different treatment modalities was comparable with p = 0.89 (Table 4). The proportion of patients with infection was comparable in cryosurgery category, diode LASER category , scalpel blade surgery and CO2 LASER category one week follow up (p=0.71). There was decrease in infection in all treatment modalities at two week follow up. The proportion of infection in at two week follow up in different treatment modalities was comparable (p=0.34) (Table 5). The incidence of bleeding was maximum (67%) in scalpel blade surgery at day of surgery as compared to other treatment modalities. The findings were statistically significant (p=0.001). There was very low incidence of bleeding in cryotherapy category, DIODE LASER and CO2 LASER and the incidence was comparable between cryotherapy category, DIODE LASER and CO2 LASER (Table 6). The proportion of patients with scar at 3 months follow up and 6 months follow up was lower in scalpel blade surgery as compared to cryosurgery category, Diode LASER category, and CO2 LASER category. The findings were significant statistically (p=0.001). There was decrease in scar in all treatment modalities at 6 month follow up. The proportion of scar at 6 month follow up in scalpel blade category was minimum as compared to cryosurgery category, Diode LASER category , and CO2 LASER category (p=0.004). The incidence of scar in cryosurgery category, Diode LASER category and CO2 LASER category was comparable at 3 months and 6 months follow up (Table 7). The proportion of patients with slough at first week follow up and second week follow up was lower in scalpel blade surgery as compared to cryosurgery category, Diode LASER category, and CO2 LASER category. The findings were significant statistically (p=0.001). There was decrease in slough in all treatment modalities at two week follow up. The proportion of slough at two week follow up in scalpel blade category was minimum as compared to cryosurgery category, Diode LASER category, and CO2 LASER category (p=0.004). The incidence of slough in cryosurgery category, Diode LASER category and CO2 LASER category was comparable at first week and second week follow up (Table 8). The proportion of patients with recurrence at 3 months follow up and 6 months follow up was lower in scalpel blade surgery as compared to cryosurgery category, Diode LASER category, and CO2 LASER category. The findings were significant statistically (p=0.001). There was decrease in recurrence in all treatment modalities at 6 month follow up. The proportion of recurrence at 6 months follow up in scalpel blade category was minimum as compared to cryosurgery category, diode LASER category, and CO2 LASER category (p=0.002). The incidence of recurrences in cryosurgery category, diode LASER category and CO2 LASER category was comparable at 3 months and 6 months follow up (Table 9).

## Discussion:

Data shows that pain at ay of surgery and bleeding was greater in conventional scalpel and blade surgery as compared to diode LASER, CO2 LASER and cryo-theraphy [16-21]. It was also observed in some studies that pain in cryotherapy was greater than LASERS. This finding is not similar to our study where pain in cryotherapy was lesser as compared to LASER [26]. The first step in treating OPMDs is to remove risk factors such smoking, using tobacco products, quitting betel quid, and reducing or quitting alcohol entirely [11- 14]. The prognosis may change significantly if smoking is completely stopped. The course of treatment will also depend on the severity of the lesion, including its location, size, and position, as well as any concomitant dysplasia [15-17]. To manage OPMDs, a variety of medication and surgical therapeutic approaches are employed. The goal of treating OPMD is to reverse or totally eradicate the changes to the oral mucosa [13-16]. Topical and systemic medications, with differing degrees of success, include vitamin A and retinoid, lycopene, ketorolac, systemic beta-carotene, topical and systemic corticosteroids, and local bleomycin in the medical line of care for OPMD.

There was low proportion of patients with oedema in cryosurgery group as compared to Diode LASER, scalpel blade surgery and CO2 LASER at one week follow up. The findings were significant statistically (0.001). There was decrease in oedema in all treatment modalities at two week follow up. The proportion of oedema in at two week follow up in different treatment modalities was comparable (p=0.89). The proportion of patients with infection was comparable . in cryosurgery category, Diode LASER category , scalpel blade surgery and CO2 LASER category at one week follow up (p=0.71). There was decrease in infection in all treatment modalities at two week follow up. The proportion of infection in at two week follow up in different treatment modalities was comparable (p=0.34). Data have showed comparable oedema in different surgical treatment modalities for OPMDs [2-6]. A study [26] has shown than oedema is greater in cryotherapy as compared to LASER unlike our data.. Although vitamin A, or retinoid, has been used medicinally, there is little evidence from research to substantiate its effectiveness in reducing the incidence of cancer transformation and relapse [4-11]. Among the surgical procedures that have been suggested for the management of OPMDs are LASER surgery and traditional surgery with a scalpel and blade [5-12]. Conventional surgeries are limited by the disease's location and extent. Cryosurgery is a simple, safe, and effective treatment for several benign and precancerous lesions of the oral cavity, as well as OPMDs [13- 15]. Lasers have many benefits over traditional surgery, such as targeted excision, simple homeostasis maintenance, and reduced postoperative pain and oedema [16-18]. While cryotherapy has proven effective in managing several medical surgeries, its application in the treatment of OPMDS remains an area of on-going investigation [20,21]. These findings are having similarity with findings of previous study where scar and recurrence at 3 months follow up and 6 months follow up was lower in scalpel blade surgery as compared to cryosurgery category, diode LASER category, and CO2 LASER category [10- 16]. Some previous studies like our study showed that the incidence of scar in cryosurgery category, diode LASER category and CO2 LASER category was comparable at 3 months and 6 months follow up [12- 18]. There were some studies in past which have shown similar results for scalpel blade surgery and cryotherapy regarding development of slough [12-16]. Randomized clinical trials have not been used to evaluate the efficacy of surgical management in preventing recurrent OPMDs and afterwards transformation into cancer, data showed that all four surgical modalities were effective in treating OPMDs.

## Conclusion:

Data shows that all four surgical modalities were effective for treatment of OPMDs. It further showed that cryotherapy seems to offer better clinically significant results than laser therapy.

## Figures and Tables

**Table 1:** Distribution of study participants

	**Treatment Modality**	**Number**
Category 1	Conventional scalpel blade surgery	15
Category 2	Diode LASER	15
Category 3	Nitrous oxide Cryotherapy	15
Category 4	CO2 LASER	15

**Table 2:** Type of OPMDs

	**No (n=60)**	**Percentage**
Homogenous leukoplakia	20	33.33
Non homogenous leukoplakia	14	23.34
Oral lichen planus	18	30
Erythroplakia	8	13.33

**Table 3:** VAS score for post-operative pain (mean ± SD) in all four treatment modalities

	**Day of surgery**	**One week follow up**	**Two week follow up**
Scalpel blade surgery	2.9± 0.13	1.6±0.02	0.7±0.04
Diode LASER	2.1± 0.01	1.4±0.04	0.5±0.01
Cryotherapy	1.6± 0.34	1.1± 0.31	0.2± 0.02
CO2 LASER	2.0± 0.12	1.5± 0.14	0.4± 0.03
P value	0.001*	0.0023*	0.67*
*indicates statistically significant difference.

**Table 4:** Incidence of oedema (%) in all four treatment modalities

	**One week follow up**	**Two week follow up**
Scalpel blade surgery	45%	3%
Diode LASER	38%	4%
Cryotherapy	25%	2%
CO2 LASER	40%	4%
P value	0.001*	0.89
*indicates statistically significant difference.

**Table 5:** Incidence of infection (%) in all four treatment modalities

	**One week follow up**	**Two week follow up**
Scalpel blade surgery	15%	0%
Diode LASER	18%	1%
Cryotherapy	15%	0%
CO2 LASER	18%	1%
P value	0.71	0.34

**Table 6:** Incidence of bleeding (%) in all four treatment modalities

	**Day of surgery**
Scalpel blade surgery	67%
Diode LASER	4%
Cryotherapy	2%
CO2 LASER	3
P value	0.001*
*indicates statistically significant difference.

**Table 7:** Incidence of scar (%) in all four treatment modalities

	**3 months**	**6 months**
Scalpel blade surgery	14%	2%
Diode LASER	38%	18%
Cryotherapy	35%	15%
CO2 LASER	38%	18%
P value	0.001*	0.004*
*indicates statistically significant difference.

**Table 8:** Incidence of slough (%) in all four treatment modalities

	**First week**	**Second week**
Scalpel blade surgery	13%	3%
Diode LASER	36%	16%
Cryotherapy	37%	17%
CO2 LASER	35%	15%
P value	0.001*	0.004*
*indicates statistically significant difference.

**Table 9:** Incidence of recurrence (%) for all four treatments

	**3 months**	**6 months**
Scalpel blade surgery	12%	4%
Diode LASER	36%	16%
Cryotherapy	32%	12%
CO2 LASER	31%	11%
P value	0.001*	0.002*
*indicates statistically significant difference.

## References

[1] Babu KG (2001). Semin in Oncol..

[2] Goharkhay K (1999). Lasers Surg Med..

[3] Zeinoun T (2001). Lasers Surg Med..

[4] Lodi G (2016). Cochrane Database Syst Rev..

[5] Frame JW (1985). J Oral Maxillofac Surg..

[6] Kardos TB (1989). Int J Oral Maxillofac Surg..

[7] Warnakulasuriya S (2016). J Oral Pathol Med..

[8] Huang Z (2015). J Craniofac Surg..

[9] Flynn MB (1988). J Surg Oncol..

[10] Van der Waal I (1997). Oral Oncol..

[11] Ben-Bassat M (1978). Br J Plast Surg..

[12] Akbulut N (2013). Eur J Dent..

[13] Napier SS (2008). J Oral Pathol Med..

[14] Sako K (1972). Am J Surg..

[15] Yeh CJ (2000). Int J Oral Maxillofac Surg..

[16] Shiu MN (2000). Br J Cancer..

[17] Lodi G (2002). J Dent Educ..

[18] Kawczyk-Krupka A (2012). Photodiagnosis Photodyn Ther..

[19] Ishii J (2003). Oral Oncol..

[20] Yu CH (2014). J Formos Med Assoc..

[21] Gupta PC (1995). Oral Dis..

[22] Ribeiro AS (2010). Int J Dent..

[23] Lin HP (2012). Head Neck..

[24] Kumar A (2013). Br J Oral Maxillofac Surg..

[25] Hong WK (1986). N Engl J Med..

[26] Natekar M (2017). J Clin Exp Dent..

